# Diagnostic reference values for sarcopenia in Tibetans in China

**DOI:** 10.1038/s41598-020-60027-0

**Published:** 2020-02-20

**Authors:** Liping Ye, Youfeng Wen, Ying Chen, Jie Yao, Xin Li, Yingying Liu, Jia Song, Zhengqi Sun

**Affiliations:** 1 Department of Pathophysiology, Jinzhou Medical University, Jinzhou, 121001 China; 2 Biological Anthropology Institute, Jinzhou Medical University, Jinzhou, 121001 China; 3 Department of Anatomy, Tibet National University, Xianyang, 712082 China

**Keywords:** Anatomy, Musculoskeletal system

## Abstract

Sarcopenia is an age-associated disease characterized by loss of muscle mass and function, but the diagnostic cutoff values remain controversial. To investigate the diagnostic cutoff values and incidence of sarcopenia in a plateau population, the limb skeletal muscle mass, gait speed and grip strength of 2318 Tibetan adults were measured according to the criteria of the Asian Working Group for Sarcopenia. We found that the diagnostic reference values for sarcopenia in the high-altitude population were significantly lower than those in the plain population, and the incidences of sarcopenia in the high-altitude population over 60 years old were 17.2% in men and 36.0% in women, which were significantly higher than those in the plain population. Our study proposes reference values for the diagnosis of sarcopenia in Tibet. We suggest that the cutoff value for sarcopenia in the plateau population should be established based on altitude. Hypoxia may be an important risk factor for sarcopenia.

## Introduction

Sarcopenia is a progressive and generalized skeletal muscle disorder involving the accelerated loss of muscle mass and function that is associated with increased adverse outcomes, including falls, functional decline, frailty, and mortality^1^. With the ageing of the population, sarcopenia has become one of the major public health problems in the world^2,3^. Since 2010, the study of sarcopenia in the field of international geriatrics has become increasingly intense, focusing on the diagnostic criteria and pathogenesis of sarcopenia. Both the European and Asian sarcopenia working groups have recommended diagnostic criteria of sarcopenia based on muscle mass, muscle strength and physical function presented by skeletal muscle mass index (SMI), handgrip strength (HS) and gait speed (GS), respectively^4,5^. Currently, the incidence of sarcopenia and the cutoff values for SMI and HS in different populations are very different. The incidence of sarcopenia is related not only to age but also to racial differences. Ethnic factors may be one of the main reasons for the differences in the incidence of sarcopenia among different populations^6–8^. The European Working Group on Sarcopenia in Older People (EWGSOP) has suggested that primary sarcopenia is mainly related to ageing and is a phenotype in the process of biological senescence. Ageing is a sequential process of genetic programming, which inevitably results in a decrease in the amount of ageing muscle, but this process is affected by the environment, lifestyle and ageing-related diseases^9^ and shows significant individual differences. Therefore, ethnic and individual differences should be taken into account in determining diagnostic reference values for sarcopenia, and the best reference values should include a wide range of races and ages. Asia is the most dense and fastest ageing region in the world, and sarcopenia will have a very large impact on the Asian population^5^. The Asian Working Group for Sarcopenia (AWGS) has recommended that a large number of investigations should be carried out to provide a basis for the establishment of diagnostic criteria for sarcopenia in Asia^10^. However, there are still no reference values for sarcopenia in the plateau population. At the same time, weight and body composition have been shown to change at high altitudes^11,12^. Therefore, we investigated the reference values of Tibetans living on the Qinghai-Xizang Plateau in China and provided cutoff value for the diagnosis of sarcopenia in the plateau population.

## Materials and Methods

### Data sources and study participants

This was a cross-sectional study. In August 2016 and 2017, the survey was carried out with a health examination in Tibet, China. Healthy Tibetans aged over 20 years participated in this study. All participants lived in Tibet, China. The sample consisted of 2318 subjects (900 men and 1418 women), and the average age was 42.33 years. A total of 1355 subjects (555 men and 800 women) were from Lhasa (altitude of 3600 metres), and 963 subjects (345 men and 618 women) were from Shigatse (altitude of 4200 metres). All participants were in good health according to clinical medical evaluations. The following subjects were excluded from the study: 1. individuals with long-term bed rest, sedentary, or extreme weight loss; 2. individuals with heart, lung, liver, kidney, brain diseases, inflammatory reaction diseases, malignant tumours, and endocrine diseases; and 3. individuals with an absorption disorder, gastrointestinal disease, or anorexia drug use. The questionnaires collected information on demographics, lifestyle, history of diseases, physical examinations, activities of daily living and physical performance. These were just to collect demographic information and sample screening. The interviewers were trained before the survey was administered. The study was approved by the Research Ethics Committee of Jinzhou Medical University in accordance with the Declaration of Helsinki. Verbal and written informed consent was obtained from all participants.

### Anthropometry

Height was measured using a portable stadiometer (HM200P, American Charder Company, America) and recorded to the nearest 0.1 cm. Body mass index (BMI) was calculated from weight and height.

### Measurement of HS

HS was measured using a handheld dynamometer based on strain gauge sensors (CAMRY EH101; Xiangshan, China) to the nearest 0.1 kg. Both hands were tested with the participant seated, elbow flexed at a 110° angle, wrist placed in a neutral position, and the interphalangeal joint of the index finger positioned at a 90° angle. Two readings were obtained for each hand, and the highest value in either hand was used for the analyses. A preliminary study was conducted to assess the reliability of the HS test using the intraclass correlation coefficient. The results showed excellent test-retest reliability of the HS test^13^.

### Measurement of GS

According to the criteria of AWGS, physical performance was assessed using the usual GS. To measure the usual GS, the participants were asked to walk a 5-metre course at their usual speed. Timing commenced when the participants started foot movement and stopped when the foot contacted the ground after completely crossing the 5-metre mark. Canes or walkers were allowed if necessary^13^.

### Measurement of appendicular skeletal muscle mass and weight

A bioelectrical impedance analyser (MC-180, Bailida, Japan) was used to measure left upper limb skeletal muscle mass (LUSM), right upper limb skeletal muscle mass (RUSM), left lower limb skeletal muscle mass (LLSM), and right lower limb skeletal muscle mass (RLSM).

### Sarcopenia cutoff value determination

According to the recommended diagnostic algorithm from the AWGS^5^, GS, HS and SMI are the primary indicators for a sarcopenia diagnosis.

#### SMI cutoff value

SMI was calculated as SMI = ASM/height^2^ (kg/m^2^). The appendicular skeletal muscle (ASM) was equal to the total muscle mass of the four limbs. Two standard deviations below the mean SMI in the young reference group (20–40 years old) was used as the SMI cutoff values determination^5^.

#### GS cutoff value

Low physical performance was defined as GS less than 0.8 m/s^5^.

#### HS cutoff value

The lower 20th percentile of handgrip strength in the study population was used as the cutoff value for low muscle strength^5^.

### Sarcopenia classification

According to the recommended diagnostic algorithm of the AWGS, the subjects with low muscle mass without an impact on muscle strength or physical performance were classified as presarcopenia, and the subjects with low muscle mass along with low muscle strength or low physical performance were considered to have sarcopenia. The subjects without low muscle mass, low HS and low GS were classified as having “no sarcopenia”^5^.

### Statistical analysis

The data, expressed as the mean ± standard deviation, were analyzed by independent t tests in the same age group. The chi-square test was used to determine whether the incidence of sarcopenia differences in the Tibetans occurred among the age groups. All analyses were performed using SPSS (ver. 20.0, IBM Company).

## Results

### Comparison of height, weight, BMI and limb skeletal muscle mass between Lhasa and Shigatse

**Figure 1:**
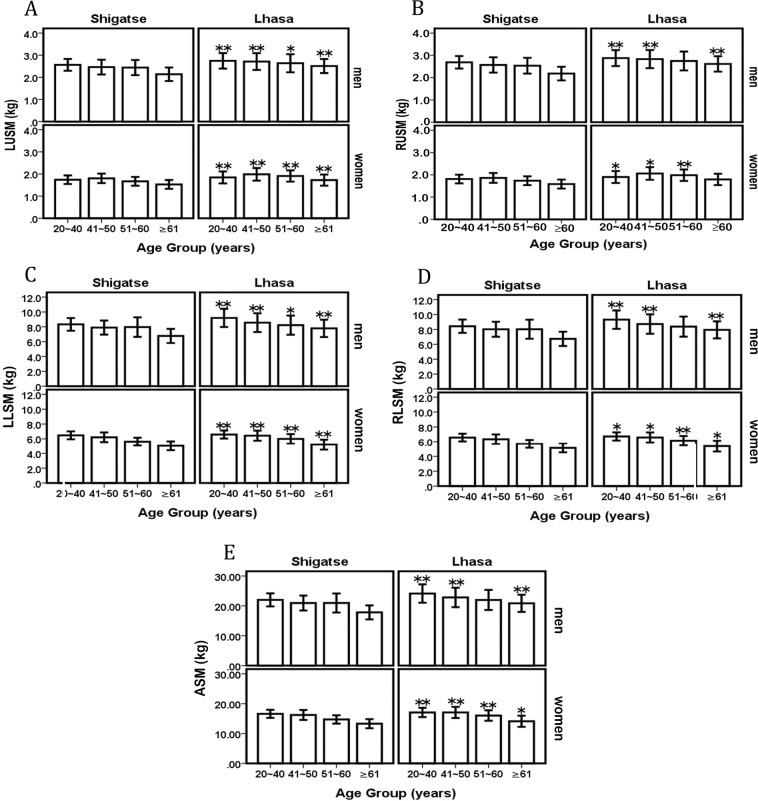
Mean changes in limb skeletal muscle mass and comparisons between Lhasa and Shigatse by age and sex. Shigatse compared with Lhasa in the same age: *P < 0.05, **P < 0.01. LUSM: left upper limb skeletal muscle mass; RUSM: right upper limb skeletal muscle mass; LLSM: left lower limb skeletal muscle mass; RLSM: right lower limb skeletal muscle mass; ASM: appendicular skeletal muscle.

Our results (Table 1) showed that, with the exception of BMI in men aged 51–60 years, the measures of height, weight and BMI in Lhasa were significantly higher than those in Shigatse (*P* < 0.05), which indicated that altitude had a certain effect on height, weight and BMI. At the higher altitude, height and weight were decreased. Within the same investigation site and for a particular sex, there were significant age differences in height, weight and BMI (*P* < 0.05), which showed that height decreased with advancing age, and weight initially increased and then decreased. From this study (Table 2; Fig. 1), we found that LUSM, RUSM, LLSM, RLSM and ASM in Lhasa were greater than those in Shigatse at the same age and sex (*P* < 0.05). This indicated that limb skeletal muscle mass decreased with increasing altitude.

**Table 1:** Anthropometry data for the Tibetan participants and comparisons between Lhasa and Shigatse by age and sex (means ± SD).

Age (years)	n		Height (m)		Weight (kg)		BMI (kg/m^2^)	
	Lhasa	Shigatse	Lhasa	Shigatse	Lhasa	Shigatse	Lhasa	Shigatse
**Women**								
20~40	303	282	160.40 ± 5.06	154.37 ± 4.79**	57.79 ± 10.19	49.89 ± 6.84**	22.41 ± 3.55	20.68 ± 2.39**
41~50	221	143	159.54 ± 5.15	154.34 ± 5.07**	63.57 ± 11.15	52.58 ± 7.09**	24.91 ± 3.89	21.15 ± 2.25**
51~60	171	123	157.69 ± 5.88	152.08 ± 5.27**	62.17 ± 9.78	50.73 ± 7.49**	24.99 ± 3.66	22.42 ± 4.10**
≥ 61	105	70	152.79 ± 7.56	149.08 ± 5.43*	57.00 ± 9.49	47.18 ± 6.31**	24.44 ± 4.06	20.46 ± 2.20**
**Men**								
20~40	221	193	172.19 ± 5.87	165.81 ± 5.80**	68.03 ± 12.31	56.91 ± 7.66**	22.92 ± 3.88	20.93 ± 2.44**
41~50	146	70	169.43 ± 6.56	165.96 ± 6.66**	67.38 ± 11.98	58.39 ± 7.72**	23.44 ± 3.80	22.05 ± 2.51**
51~60	118	53	168.20 ± 5.51	165.73 ± 6.43*	66.65 ± 11.86	61.36 ± 11.65*	23.48 ± 3.55	21.91 ± 2.94
≥ 61	70	29	165.31 ± 6.70	162.08 ± 5.95*	65.09 ± 9.72	53.83 ± 7.11**	23.78 ± 3.06	21.18 ± 2.39**

Shigatse compared with Lhasa at the same age: *P < 0.05, **P < 0.01. BMI: body mass index.

**Table 2:** Characteristics of limb skeletal muscle mass and comparisons between Lhasa and Shigatse by age and sex (means ± SD, kg).

Age (years)	n		LUSM		RUSM		LLSM		RLSM		ASM	
	Lhasa	Shigatse	Lhasa	Shigatse	Lhasa	Shigatse	Lhasa	Shigatse	Lhasa	Shigatse	Lhasa	Shigatse
**Women**												
20~40	303	282	1.84 ± 0.27	1.74 ± 0.20**	1.90 ± 0.27	1.81 ± 0.19*	6.58 ± 0.54	6.47 ± 0.53**	6.72 ± 0.55	6.57 ± 0.52*	17.05 ± 1.55	16.59 ± 1.35**
41~50	221	143	1.98 ± 0.28	1.80 ± 0.21**	2.06 ± 0.28	1.86 ± 0.22*	6.43 ± 0.68	6.21 ± 0.66**	6.58 ± 0.68	6.34 ± 0.65*	17.05 ± 1.87	16.21 ± 1.67**
51~60	171	123	1.91 ± 0.26	1.67 ± 0.20**	1.98 ± 0.26	1.74 ± 0.20**	5.99 ± 0.65	5.61 ± 0.54**	6.14 ± 0.65	5.70 ± 0.54**	16.02 ± 1.75	14.72 ± 1.39**
≥ 61	105	70	1.72 ± 0.25	1.53 ± 0.20**	1.79 ± 0.26	1.58 ± 0.20	5.20 ± 0.69	5.04 ± 0.61**	5.39 ± 0.74	5.14 ± 0.60*	14.10 ± 1.87	13.29 ± 1.52*
**Men**												
20~40	221	193	2.74 ± 0.35	2.56 ± 0.27**	2.88 ± 0.36	2.69 ± 0.28**	9.19 ± 1.23	8.32 ± 0.85**	9.31 ± 1.24	8.42 ± 0.89**	24.13 ± 3.07	22.00 ± 2.19**
41~50	146	70	2.71 ± 0.38	2.46 ± 0.33**	2.83 ± 0.41	2.57 ± 0.34**	8.56 ± 1.27	7.89 ± 0.94**	8.72 ± 1.31	8.02 ± 1.01**	22.82 ± 3.27	20.94 ± 2.50**
51~60	118	53	2.64 ± 0.41	2.44 ± 0.34*	2.75 ± 0.42	2.54 ± 0.35	8.22 ± 1.29	7.96 ± 1.32*	8.37 ± 1.35	8.02 ± 1.28	21.98 ± 3.37	20.96 ± 3.19
≥ 61	70	29	2.51 ± 0.32	2.14 ± 0.31**	2.62 ± 0.35	2.18 ± 0.31**	7.79 ± 1.17	6.77 ± 0.95**	7.94 ± 1.15	6.73 ± 0.96**	20.85 ± 2.87	17.82 ± 2.35**

Shigatse compared with Lhasa in the same age, *P < 0.05, **P < 0.01. LUSM: left upper limb skeletal muscle mass; RUSM: right upper limb skeletal muscle mass; LLSM: left lower limb skeletal muscle mass; RLSM: right lower limb skeletal muscle mass; ASM: appendicular skeletal muscle.

### Sarcopenia diagnostic cutoff values for Tibetan individuals

**Figure 2:**
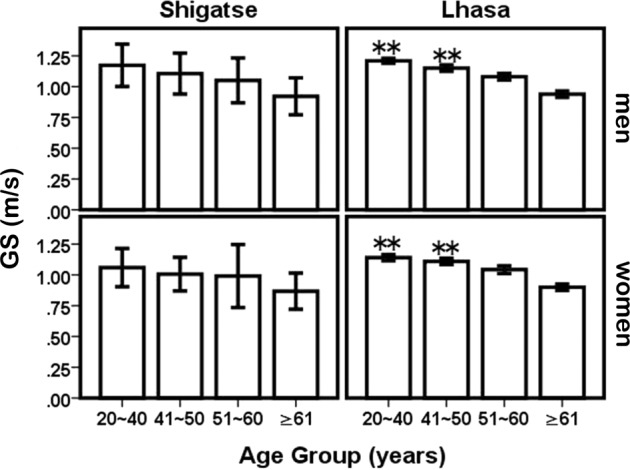
Mean changes in GS and comparisons between Lhasa and Shigatse by age and sex. Shigatse compared with Lhasa in the same age: *P < 0.05, **P < 0.01. GS: gait speed.

The results of HS, GS and SMI measurements are shown in Table 3. We found that HS, GS and SMI decreased with age. GS in Lhasa for those aged from 20 to 50 years was significantly greater than that in Shigatse of the same sex (Table 3; Fig. 2). This suggested that at the higher altitude, physical performance was slower. Especially for Shigatse Tibetan participants aged over 61 years, the GS value (0.87 m/s) was close to 0.8 m/s, which showed that those subjects had sarcopenia.

**Table 3:** HS, GS and SMI data and comparisons between Lhasa and Shigatse by age group and sex (means ± SD).

Age (years)	n		GS (m/s)		HS (kg)		SMI (kg/m^2^)	
	Lhasa	Shigatse	Lhasa	Shigatse	Lhasa	Shigatse	Lhasa	Shigatse
**Women**								
20~40	303	282	1.14 ± 0.13	1.06 ± 0.16**	23.30 ± 5.14	15.27 ± 5.80**	6.62 ± 0.50	6.96 ± 0.48**
41~50	221	143	1.11 ± 0.12	1.01 ± 0.14**	22.97 ± 5.38	13.76 ± 4.66**	6.69 ± 0.56	6.80 ± 0.57
51~60	171	123	1.04 ± 0.17	0.99 ± 0.26	19.08 ± 4.60	11.68 ± 4.27**	6.43 ± 0.52	6.36 ± 0.49
≥ 61	105	70	0.91 ± 0.21	0.87 ± 0.15	14.33 ± 4.38	11.14 ± 3.79**	6.03 ± 0.58	5.97 ± 0.50**
**Men**								
20~40	221	193	1.21 ± 0.15	1.17 ± 0.17**	37.99 ± 7.35	24.91 ± 8.69**	8.13 ± 0.88	8.00 ± 0.61
41~50	146	70	1.15 ± 0.12	1.11 ± 0.17**	35.10 ± 7.95	19.74 ± 8.47**	7.94 ± 0.96	7.59 ± 0.66**
51~60	118	53	1.08 ± 0.16	1.05 ± 0.18	31.63 ± 7.15	16.61 ± 7.59**	7.74 ± 0.90	7.61 ± 0.93*
≥ 61	70	29	0.94 ± 0.14	0.92 ± 0.15	24.46 ± 7.38	13.27 ± 6.74**	7.61 ± 0.81	6.78 ± 0.73**

Shigatse compared with Lhasa at the same age: **P < 0.01. GS: gait speed; HS: handgrip strength; SMI: skeletal muscle mass index.

**Figure 3:**
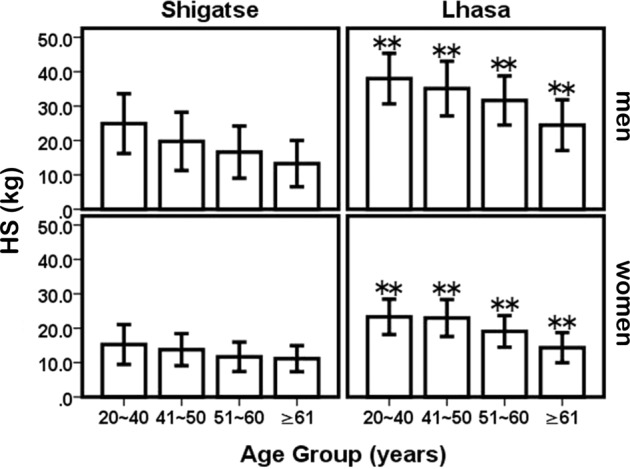
Mean changes in HS and comparisons between Lhasa and Shigatse by age and sex. Shigatse compared with Lhasa in the same age: *P < 0.05, **P < 0.01. HS: handgrip strength.

HS in Lhasa was greater than that in Shigatse at the same age and sex (P < 0.05) (Table 3; Fig. 3). According to the AWGS strategy, the lower 20th percentile of HS in the study population was used as the cutoff value for low muscle strength. Thus, the cutoff values for HS in Lhasa were 26.7 kg (men) and 15.8 kg (women), and in Shigatse, these values were 13.3 kg (men) and 8.9 kg (women).

**Figure 4:**
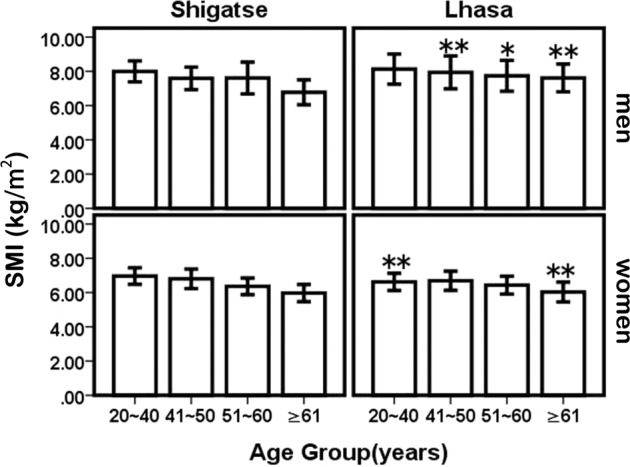
Mean changes in SMI and comparisons between Lhasa and Shigatse by age and sex. Shigatse compared with Lhasa in the same age: *P < 0.05, **P < 0.01. SMI: skeletal muscle mass index.

The reference value of SMI refers to the mean SMI value in the study population aged 20 to 40 years old. From our results (Table 3; Fig. 4), there was a significant difference in SMI between Lhasa and Shigatse in women aged 20–40 years (P < 0.05). However, there was no difference in men. Therefore, the common SMI reference value of 8.07 ± 0.77 kg/m^2^ was used to diagnose sarcopenia in men. In women, the SMI reference values in Lhasa and Shigatse were 6.62 ± 0.50 kg/m^2^ and 6.96 ± 0.48 kg/m^2^, respectively. Thus, in men, the SMI cutoff value was 6.53 kg/m^2^; in women, the cutoff values in Lhasa and Shigatse were 5.62 kg/m^2^ and 6.0 kg/m^2^, respectively.

### Incidence of sarcopenia among the Tibetan participants

According to the methods of AWGS and the cutoff values obtained in this study, the incidence of sarcopenia in the Tibetan participants was determined as shown in Table 4. Among Tibetans over the age of 40 years, the incidence of sarcopenia in women was 13.3% and that in men was 8.6%. The incidence of sarcopenia increased significantly with age, especially in women, where these values increased from 3.8% to 36% across ages. The incidence of sarcopenia was significantly higher in Shigatse than in Lhasa (*P* < 0.05).

**Table 4:** Incidence of sarcopenia in Tibetans and comparisons among the different ages by region.

Age (years)	Lhasa					Shigatse					Tibet				
	Sarcopenia					Sarcopenia						Sarcopenia			
	NO (%)	YES (%)	n1	χ^2^	*P*	NO (%)	YES (%)	n2	χ^2^	*P*	Total	NO (%)	YES (%)	χ^2^	*P*
**Women**															
41~50	216 (97.7)	5 (2.3)	221	61.76	0.000	134 (93.7)	9 (6.3)	143	56.419	0.000	364	350 (96.2)	14 (3.8)	107.01	0.000
51~60	165 (96.5)	6 (3.5)	171			95 (77.2)	28 (32.9)	123			294	260 (88.4)	34 (11.6)		
≥61	78 (74.3)	27 (25.7)	105			34 (48.6)	36 (51.4)	70			175	112 (64)	63 (36.0)		
total	459 (92.4)	38 (7.6)	497			263 (78.3)	73 (21.7)	336			833	722 (86.7)	111 (13.3)		
**Men**															
41~50	138 (94.5)	8 (5.5)	146	1.654	0.437	66 (94.3)	4 (5.7)	70	19.584	0.000	216	204 (94.4)	12 (5.6)	11.963	0.003
51~60	108 (91.5)	10 (8.5)	118			50 (94.3)	3 (5.7)	53			171	158 (92.4)	13 (7.6)		
≥61	63 (90.0)	7 (10.0)	70			19 (65.5)	10 (34.5)	29			99	82 (82.8)	17 (17.2)		
total	309 (92.5)	25 (7.5)	334			135 (88.8)	17 (11.2)	152			486	444 (91.4)	42 (8.6)		

n1, n2 and n3 were the number of subjects of different age groups by sex and region, respectively. χ^2^ and P were the comparison of Incidence on sarcopenia.

## Discussion

Hypoxic environments have been shown to influence a person’s body composition (e.g., reductions in body weight, fat-free mass, fat mass, muscle mass and/or body water)^14^. Tibetans live on the Qinghai-Xizang Plateau, which is a typical plateau environment. Our results (Tables 1 and 2) showed that the height, weight, BMI and limb skeletal muscle mass of Tibetans were reduced with increasing altitude. This was in accordance with a previous study^15–17^.

**Figure 5:**
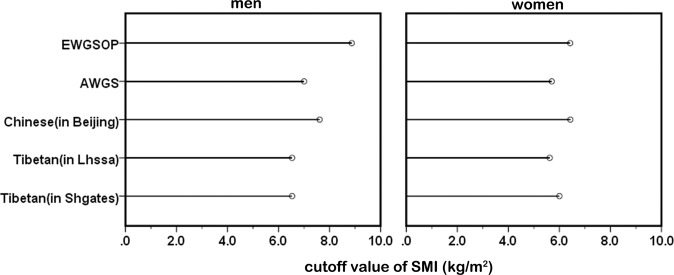
Comparison of SMI reference values among the different populations. SMI: skeletal muscle mass index.

**Figure 6:**
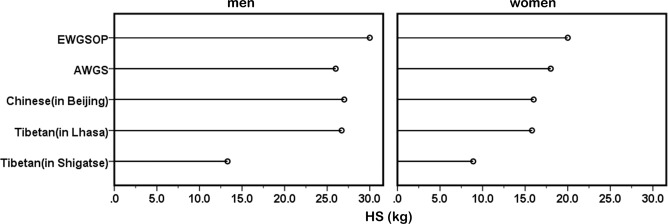
Comparison of HS reference values among the different populations. HS: handgrip strength.

The AWGS has suggested that the cutoff values for sarcopenia may be different because of ethnicities, body size, lifestyles, and cultural backgrounds^5^. To date, there are no specific cutoff values for the plateau population. In this study, we established cutoff values for sarcopenia in Tibetans living at altitudes over 3000 metres. By comparison, it was found that the cutoff values for SMI and HS in Tibetans were lower than those suggested by the EWGSOP and AWGS and for the Chinese population in Beijing (Table 5; Figs. 5 and 6). The cutoff values from the EWGSOP and AWGS and for the Chinese population in Beijing were not applicable to Tibetans.

**Table 5:** Diagnostic cutoff value for sarcopenia in different populations.

	SMI (kg/m^2^)		HS (kg)	
	men	women	men	women
EWGSOP^4^	<8.87	<6.42	<30	<20
AWGS^5^	<7.0	<5.7	<26	<18
Chinese (in Beijing)^33^	<7.61	<6.43	<27	<16
Tibetan (in Lhasa)	<6.53	<5.62	<26.7	<15.8
Tibetan (in Shigatse)	<6.53	<6.0	<13.3	<8.9

SMI: skeletal muscle mass index; HS: handgrip strength.

There was no significant difference in SMI reference values between Lhasa and Shigatse in Tibetan men; thus, the common reference value 8.07 ± 0.77 kg/m^2^ was used. However, in Tibetan women, the SMI reference values in Shigatse were greater than those in Lhasa, and the respective reference values should be used. The reason that the SMI reference values in Shigatse were higher than those in Lhasa may be that (1) the participants in Shigatse were from agricultural and pastoral areas, and the participants in Lhasa were from towns, which led to the ASM proportion of the weight being higher in Shigatse (33.93%) than in Lhasa (29.50%); (2) the height of the Tibetans in Shigatse (154.37 cm) was lower than that of those in Lhasa (160.40 cm). We suggest that the cutoff values for sarcopenia in the plateau population should be established according to altitude, such as moderate altitude: 1500–3500 m; high altitude: 3500–5300 m; extreme altitude: >5300 m.

Studies have shown that in plateau environments, weight loss is mainly caused by loss of fat mass^18^, while in pathological hypoxia (such as chronic obstructive pulmonary disease), weight loss is mainly due to losses in fat-free mass, such as muscle^19^. In the process of ageing, the structural and functional changes of skeletal muscles are similar to those of hypoxia^11^, which has been considered to be the cause of muscle atrophy and decreased contractility^6^. Therefore, some researchers believe that hypoxia may be the main cause of sarcopenia during ageing. Kayser’s study showed that body weight loss was initially due to water loss, followed by loss of fat mass and muscle mass and that more body weight was lost than fat^20^. Other studies have shown that the incidence of sarcopenia in patients with chronic obstructive pulmonary disease was higher than that in normal people^21^. The results showed that the skeletal muscle mass of the extremities in individuals in Shigatse (4200 m) was lower than that in those in Lhasa city (3600 m), and the incidence of sarcopenia in Shigatse was greater than that in Lhasa. The results indicated that hypoxia on the plateau has an important effect on the mass and function of skeletal muscle. Based on the comparison of the incidence of sarcopenia (Table 6), the incidence of sarcopenia in the plateau population was higher than that in the plain population. It has been reported that the incidences of sarcopenia in Beijing adults aged 60 years or older were 11.3% for men and 18.7% for women^22^, and in Taiwan, they were 8.2% for men and 6.5% for women^23^. However, in this study, we found that the incidences of sarcopenia in Tibetans were 17.2% for men and 36.0% for women. The incidences of sarcopenia in Tibet were higher than those in other parts of China and Asia. Compared to Beijing and Taiwan, the odd ratios were 1.42 (for men) and 3.65 (for women) and 7.53 (for men) and 26.00 (for women), respectively, which indicated that hypoxia might be a risk factor for sarcopenia. Hypoxia may be the main cause of sarcopenia in the ageing process. In the ageing process, changes in skeletal muscle structure and function are similar to those observed in hypoxia^24^. Studies have shown that for every 1000 feet above sea level, the maximum oxygen consumption decreases by 3.2%, which is similar to the change in maximum oxygen consumption during ageing^25^. Chronic hypoxia exposure caused by a high-altitude environment accelerates the decomposition of skeletal muscle. At the same time, protein synthesis was inhibited, resulting in a decline in skeletal muscle mass^26^. Hypoxia can increase the concentration of hormones in the blood and inhibit the endocrine response^6^, while changes in endocrine function (such as testosterone, oestrogen, adrenocortical hormone, insulin, etc.) and skeletal muscle content and function are closely related to the occurrence of sarcopenia^8^. Wandrag L^27^ showed that FFM loss was associated with increased levels of biomarkers related to the NO pathway (e.g., nitrite), oxidative stress (e.g., 4-HNE), inflammation (e.g., IL-6) and metabolic efficiency (e.g., GLP-1 and insulin) in a hypobaric hypoxic environment. These factors may be the reason for the high incidence of sarcopenia in the plateau environment.

**Table 6:** Comparison of the incidence of sarcopenia in the population over 60 years of age in different areas.

Sex	Sarcopenia	Tibet (%)	Beijing (%)^22^	Taiwan (%)^23^
Men	Yes	17.2	11.3	8.2
	No	82.8	88.7	91.8
Women	Yes	36.0	18.7	6.5
	No	64	81.3	93.5

OR (Beijing, men) = 1.42. OR (Beijing, women) = 3.65.

OR (Taiwan, men) = 7.53. OR (Taiwan, women) =26.00.

One of the limitations of this study was that in the plateau environment, the reliability of bioimpedance analysis (BIA) needs further research to be verified. Dual-energy X-ray (DXA) is the most accurate equipment to measure skeletal muscle mass. The medical and economic conditions are poor in Tibet, and therefore, there is little DXA equipment available in Tibet, and it would not have been convenient to carry. However, because the Tibetans lived in a scattered dispersal pattern, the BIA was suitable for measuring skeletal muscle mass in this study. Some studies have shown that there is a good correlation between BIA results under standard conditions and MRI prediction results^28^. However, the use of BIA for the assessment of body composition in plateaus has been challenged, as changes in hydration may influence the accuracy of the measurements^29,30^. The second limitation of this study was that the sample size for those over the age of 61 was small. According to China’s sixth census data, Tibet’s population that is over 65 years old accounts for only 5.09%, and the average life expectancy is 68.2 years^31^. This was consistent with studies that have shown that when the altitude was higher, life expectancy was shorter^32^. This led to difficulty in investigating individuals over 61 years old, especially in Shigatse, which is located 4200 metres above sea level. The insufficiency of the sample size of those over 61 years old may have led to a certain deviation in the incidence of sarcopenia in Tibet. At the same time, among those who were 51–60 years old, our results showed that the incidence of sarcopenia (32.9%) in Shigatse (above 4200 metres) was significantly higher than that (3.5%) in Lhasa (above 3600 metres). Therefore, we suggest that the investigation of the incidence of sarcopenia in plateaus above 4000 metres should be carried out from the age of 50.

Our findings provide reference values and the incidence of sarcopenia in Tibetans. We suggest that the cutoff values for sarcopenia in plateau populations should be established based on the altitude. Hypoxia may be an important risk factor for sarcopenia, but the mechanism is to be further studied.
